# Therapeutic effect of demethylated hydroxylated phillygenin derivative on *Helicobacter pylori* infection

**DOI:** 10.3389/fmicb.2023.1071603

**Published:** 2023-05-19

**Authors:** Ru-Jia Li, Jia-yin Xu, Xue Wang, Li-juan Liao, Xian Wei, Ping Xie, Wen-yan Xu, Zhen-yi Xu, Shuo-hua Xie, Yu-ying Jiang, Liang Huang, Lu-yao Wang, Gan-rong Huang, Yan-Qiang Huang

**Affiliations:** ^1^Key Laboratory of the Prevention and Treatment of Drug Resistant Microbial Infecting (Youjiang Medical University for Nationalities), Education Department of Guangxi Zhuang Autonomous Region, Baise, China; ^2^Clinical Laboratory of 980 Hospital of PLA Joint Logistics Support Force (Bethune International Peace Hospital), Shijiazhuang, Hebei, China

**Keywords:** *Helicobacter pylori*, phillygenin, demethyl hydroxylation, derivatives, therapeutic effect

## Abstract

Modifying and transforming natural antibacterial products is a novel idea for developing new efficacious compounds. Phillygenin has an inhibitory effect on *H. pylori*. The aim of the present study was to prepare a phillygenin derivative (PHI-Der) through demethylation and hydroxylation. The minimum inhibitory concentration of 18 strains of *H. pylori* from different sources was 8–32 μg/mL *in vitro*, and the activity increased 2–8 times than that of phillygenin. PHI-Der could significantly inhibit the colonization of *H. pylori in vivo*, reduce the inflammatory response, and promote the repair of inflammatory damage. Further, we used SwissTargetPrediction to predict that its main targets are *ALOX5*, *MCL1*, and *SLC6A4*, and find that it can inhibit bacterial biofilm formation and reduce bacterial infection of cells. It can enhance the intracellular oxidative capacity of *H. pylori* to inhibit *H. pylori* growth. Further, it could prevent the oxidation of *H. pylori*-infected cells and reduce the inflammatory response, which plays a role in protection. In conclusion, compared to phillygenin, PHI-Der had better antibacterial activity and was more effective in treating *H. pylori* infection. It has characteristics of high safety, specificity, resistance to drug resistance and better antibacterial activity than phillygenin, it’s a good antioxidant for host cells.

## Graphical Abstract

The figure shows the hypothesis of the mechanism of phillygenin derivatives (PHI-Der). A is PHI-Der in the treatment of gastritis mice; B is the inhibitory effect of PHI-Der on *H. pylori* biofilm; C is PHI-Der that enhance the oxidation of *H. pylori*; D is PHI-Der reduce the oxidative effect and the expression of inflammatory factors in *H. pylori-*infected cells. 
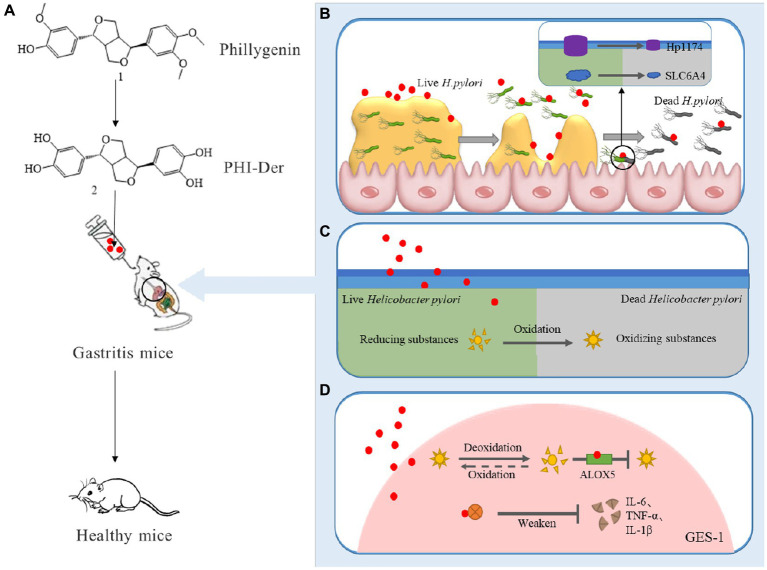

## 1. Introduction

*Helicobacter pylori* is a spiral-shaped, micro-anaerobic, Gram-negative bacteria that requires harsh growth conditions (Martin Nuñez Gracia et al., 2021). Currently, antibiotics are mainly used for treatment. However, with the widespread use and abuse of antibiotics, the resistance rate of *H. pylori* has gradually increased (Morilla et al., 2019). Therefore, the World Health Organization in 2017 listed *H. pylori*, which is resistant to clarithromycin, as one of the 12 pathogens that urgently require the development of new antibiotics (Branswell, 2017). An effective way to prepare new antibacterial drugs is to find active ingredients from natural products (natural plants, microbial secondary metabolites, marine organisms) and then modify and transform them to form derivatives (Adjissi et al., 2022). Phillygenin belongs to the class of diepoxylignans, in which two molecular phenylpropanoid side chains are connected to each other to form two epoxy structures. Phillygenin has various functions, including anti-inflammatory, anti-tumor, and antibacterial (Siqi et al., 2021; Yufeng and Peng, 2021; Wang J. et al., 2022). Preliminary research in our laboratory showed that phillygenin had a good inhibitory effect on *H. pylori*, although not comparable to the level of antibiotics. Therefore, the aim of this study was to modify and transform phillygenin to improve its antibacterial activity and explore its mechanism of action to provide an experimental basis for better utilization of phillygenin and its derivatives.

## 2. Materials and methods

### 2.1. Recovery and culture of strains

We extracted *H. pylori* strains containing the preservation solution at −80°C. Standard strains 26,695, NSH57, MSD132, and G27 were donated by Bi Hongkai from the Nanjing Medical University, and clinical strains HPBS001–HPBS016 were isolated at our laboratory. *H. pylori* strains were cultured on the Columbia blood agar plate (OXOID, UK) or in the brain-heart infusion (BHI, OXOID, UK) broth medium containing 10% serum (Pingrui, China) and placed under microaerophilic (85% nitrogen, 5% oxygen, 10% carbon dioxide; model CB160; Binder, Germany) conditions at 37°C for 3 days. Bacterial species other than *H. pylori* were cultured on nutrient agar or Luria–Bertani plates at 37°C for 1–2 days. Supplementary Table S1 shows the information of *Staphylococcus aureus* and other information.

### 2.2. Synthesis, identification and prediction of physicochemical properties of PHI-Der

First, we dissolved phillygenin (7 mg) in dry dichloromethane (5 mL). Second, we slowly added boron tribromide in dichloromethane dropwise into phillygenin at −50°C (5.0 eq, 125 mg in 5 mL dichloromethane) and placed it under ice bath for 1 h. Third, we quenched the reaction with 1 mL methanol and concentrated the reaction solution. Fourth, we performed high-performance liquid chromatography for purification (acetonitrile in water, 5–95%; flow rate, 2 mL/min, trifluoroacetic acid, 0.1%; room temperature 25°C ± 5°C) to obtain PHI-Der at a yield of 45%. After Fourier-transform infrared analysis, mass spectrometry and hydrogen and carbon nuclear magnetic resonance (NMR), the structure of PHI-Der was determined. The 3D structure of PHI-Der was visualized using ChemDraw3D. The SMILES structure was uploaded to PaddleHelix^1^ to predict the physical and chemical properties (Fang et al., 2022).

### 2.3. Microdilution to detect the minimum inhibitory concentration (MIC)

In the first well of the 96-well plate, we add 173.6 μL of the culture medium and 6.4 μL of PHI-Der (4 mg/mL) and diluted them sequentially. Negative (sterile, only medium and drug) and positive (no drug, only medium and bacteria) wells were used as controls. Bacteria growing in the logarithmic phase on the solid plate were used to create a bacterial suspension with the corresponding medium. Optical density at 600 nm (OD_600_) of *H. pylori* was adjusted to 1.0 × 10^7^ colony forming unit (CFU)/mL. OD_600_ of the remaining bacteria and the fungus were adjusted to 1 × 10^6^ and 5 × 10^3^ CFU/mL, respectively, and 10 μL was added to each well. Cultivation was performed for 24–72 h before further analyses (Wang Y. et al., 2022).

### 2.4. Detection of the minimum bactericidal concentration (MBC) using microdilution and the spread plate method

We added 173.6 μL of the culture medium and 6.4 μL of PHI-Der (4 mg/mL) to the first well of the 96-well plate. The wells were sequentially folded and diluted. The wells of phosphate-buffered saline (PBS; Sangon Biotech, China) were used as positive controls. *H. pylori* G27 grown in the logarithmic phase on the solid plate was used to create a bacterial suspension in the BHI medium, and OD_600_ of the bacterial solution was adjusted to 1 × 10^7^ CFU/mL. We added 10 μL of the bacterial solution to each well and performed culture in a three-gas incubator. After PHI-Der had been used for a certain time period (such as 2 h), we diluted (100 times, 1,000 times, and so on) the bacterial liquid, spread it on Columbia agar plates, and performed culture in a three-gas incubator for 4–5 days. We calculated the number of bacteria growing on the agar plate. The drug concentration at which the number of bacteria was inhibited by 99.9% was considered to be MBC.

### 2.5. Detection of drug resistance of PHI-Der

Long-term contact of bacteria with low doses of drugs causes changes in the medicinal chemical processes of the cells themselves, so that bacteria gradually tolerate drugs. The drug resistance of PHI-Der was detected with the *H. pylori* G27 strain. MICs of metronidazole and PHI-Der were 2 and 16 μg/mL, respectively. We used one-fourth of MIC to induce the strain, which was detected every 3 days over 24 days of induction. The induction concentration was adjusted with changes in MIC. For example, when MIC of metronidazole changed to 16 μg/mL, the induction concentration was adjusted to 4 μg/mL.

### 2.6. Cytotoxicity detection of PHI-Der

GES-1 and BGC823 (KeyGen Biotech, Nanjing, China) cell suspensions were adjusted to 1 × 10^5^. We inoculated 100 μL/well into 96-well plates and replicated three same samples. Incubation was performed at 37°C for 24 h. The final concentrations of PHI-Der were 200, 150, 100, 50, and 0 μg/mL. Subsequently, incubation was performed at 37°C for 24 h. We added 10 μL of Cell Counting Kit-8 (CCK-8; Beyotime, China) to each well, tapped to mix, and incubated for 4 h. Finally, we measured the absorbance at 450 nm.

### 2.7. Animal toxicity of PHI-Der

We purchased 6–8-week-old specific-pathogen-free (SPF) C57BL/6 mice from Changsha Tianqin Biotechnology Co., Ltd. (license number: SYXK Gui2017-0004; animal experiment ethics committee approval number: NO.2019112501). Twenty animals were randomly divided into administration and negative control groups, with ten animals in each group (Not infected with Hp), and raised in SPF environment. The administration group was administered with 10 times the therapeutic dose daily for 3 consecutive days, while the negative control group was administered with PBS solution. The mice were weighed consecutively for 7 days, starting 1 day before administration. Three days after drug withdrawal, the mice in each group were weighed, and the average body weight was calculated. Blood was collected from the eyeball, and the mice were sacrificed through dislocation and neck dislocation. Stomach, kidney, liver, and spleen tissues were obtained for pathological sectioning and hematoxylin and eosin (H&E) staining.

### 2.8. Construction of an animal model of acute gastritis to detect the inhibitory effect of PHI-Der on *Helicobacter pylori*

PHI-Der, omeprazole (Sigma-Aldrich, Germany), amoxicillin (Sigma–Aldrich, Germany), and clarithromycin (Sigma-Aldrich, Germany) were dissolved and diluted to 10 mg/mL. We successfully modeled (HPBS001) 6–8-week-old SPF C57BL/6 mice and divided them into four groups: the omeprazole + amoxicillin + clarithromycin group (omeprazole, 138.2 mg/kg; amoxicillin, 28.5 mg/kg; clarithromycin, 14.3 mg/kg), the omeprazole + PHI-Der (28 mg/kg) group, the omeprazole + PHI-Der (7 mg/kg) group, and the PBS/negative control group. Each group comprised of ten mice. The negative control group comprised of ten mice not infected with *H. pylori*. Drugs were administered daily for 3 consecutive days. Two days after drug withdrawal, blood was collected from the eyes of the mice in the infected group, and the mice were sacrificed through cervical dislocation. Gastric tissues were collected and crushed. A portion of gastric tissues was paraffin-sectioned and stained with H&E. Terminal deoxynucleotidyl transferase biotin-dUTP nick end labeling immunohistochemistry and immunofluorescence histochemistry were performed, and ImageJ was used to quantify the fluorescent signal of the immunohistochemical staining done on the tissue samples.

### 2.9. Target prediction

PHI-Der targets were obtained from the SwissTargetPrediction database.^2^ The targets duplicated with “*Helicobacter pylori* infection” were screened from the GeneCards database as potential targets of PHI-Der against *H. pylori* infection. The target interaction relationship was obtained from the STRING database.^3^ Potential targets were imported. The species selected for the study was “*Homo sapiens*.”

### 2.10. Molecular docking

The three-dimensional structure of PHI-Der was imported into Discovery Studio. We used the module for preparing ligands in molecules to process small molecules. The module mainly minimizes the energy of small molecules and yields CHARMM force fields. The crystal structure of the target protein corresponding to the core target was downloaded from the Protein Data Bank website.^4^ The protein was preprocessed using Discovery Studio to remove water molecules, hydrogenation, and charges, and the crystal structure was extracted. PyMOL was used to visualize the processed protein. AutoDock Vina performed molecular docking. PyMOL was used to combine the docking results to form a complex, and Discovery Studio was finally used for the interaction analysis and visualization of docking.

### 2.11. Inhibition experiment of PHI-Der in biofilms

The OD of the *H. pylori* G27 bacterial suspension was adjusted to 0.1, and the biofilm formed under microaerophilic conditions for 3 days. PHI-Der was added at concentrations of 128, 64, 32, and 16 μg/mL for 24 h. Its anti-biofilm activity was evaluated using crystal violet (Macklin, China) and Alamar blue (Solarbio, China) staining. The biofilm protein content was determined using the bicinchoninic protein concentration assay kit (Beyotime, China).

### 2.12. Oxidation effect of PHI-Der on *Helicobacter pylori*

*Helicobacter pylori* G27 was cultured to the logarithmic phase. The bacterial suspension was adjusted to 1 × 10^7^ CFU/mL. We added 10 μM of 2′,7′-dichlorofluorescein diacetate (DCFH-DA, Biyuntian, S0033S) to the bacterial suspension, and cultivate in a three-gas incubator for 1 h. The cocultured bacterial solution was washed twice with PBS buffer, in order to remove excess DCFH-DA. Phillygenin and PHI-Der were added at concentrations of 32 μg/mL each. PBS and Rosup were used as negative and positive controls, respectively. After 2 h of action, 15 μL of the solution was added dropwise onto the slides and observed under a fluorescence microscope.

### 2.13. Detection of the antioxidative activity of PHI-Der in GES-1 cell lines

Bacterial adhesion cell experiment GES-1(Rhodamine staining) cells were cultured in RPMI1640 medium containing 10% FBS without antibiotics, the *H. pylori* suspension was collected and labeled with SYTO9 for 15 min, and then treated with different drug concentrations and co-cultured with the cells for 2 h. The plate is placed under a fluorescence microscope for observation.

GES-1 cells were seeded (5 × 10^4^ cells/well) and grown in 96-well plates until 70% confluence. PHI-Der was incubated with cells for 2 h at 37°C. The cells were washed with PBS, incubated with 20 μM of DCFH-DA for 30 min at 37°C, and washed twice with PBS to remove the unabsorbed probe. Suspensions in the serum and antibiotic free medium were infected with *H. pylori* (1 × 10^8^ CFU/mL). Reactive oxygen species (ROS) levels were measured for 180 min using a fluorescence microplate Synergy HT reader with λex of 485 nm and λem of 530 nm.

### 2.14. Reverse transcription quantitative polymerase chain reaction (RT-qPCR)

*Helicobacter pylori* G27 was cultured to the logarithmic phase, and the bacterial suspension was adjusted to 1 × 10^8^ CFU/mL, then add PHI-Der. The cells were plated to a 70% fit and divided into the cell group, drug action group, *H. pylori* infection group, and drug action following *H. pylori* infection group. Pellets were collected using centrifugation. RNA was extracted using the TRIzol reagent (Takara, China) and reverse-transcribed into complementary DNA using a reverse transcription kit (MONPURE, China). RT-qPCR was performed using LightCycler according to the kit (MONPURE, China), with pre-denaturation at 95°C for 30 s, denaturation at 95°C for 10 s, and annealing and extension at 60°C for 30 s for 40 cycles. Supplementary Table S2 shows the primers (Sangon Biotech, Shanghai). Changes in transcript levels were determined by applying a relative quantification (2^–∆∆CT^ method) approach, with 16S ribosomal RNA amplicons used as an internal control for data normalization.

### 2.15. Statistical analysis

Data are expressed as mean ± standard deviation. Statistical analyses were performed using SPSS 25.0. One-way analysis of variance was performed, and *p* < 0.05 was considered statistically significant.

## 3. Results

### 3.1. Preparation of PHI-Der

**Figure 1:**
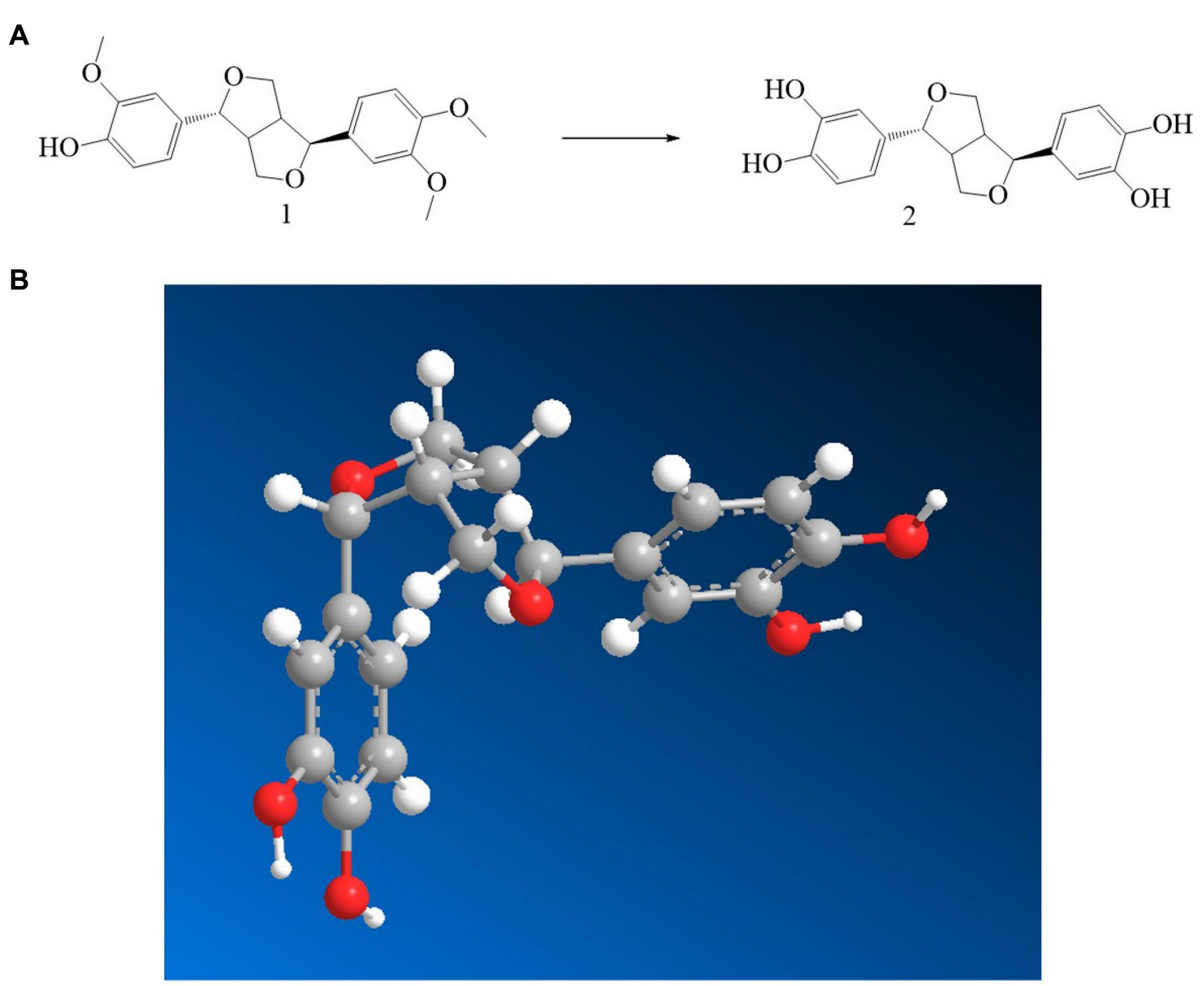
Synthesis and structure diagram of PHI-Der. **(A)** Preparation circuit of PHI-Der. **(B)** Three-dimensional structure of PHI-Der.

**Figure 2:**
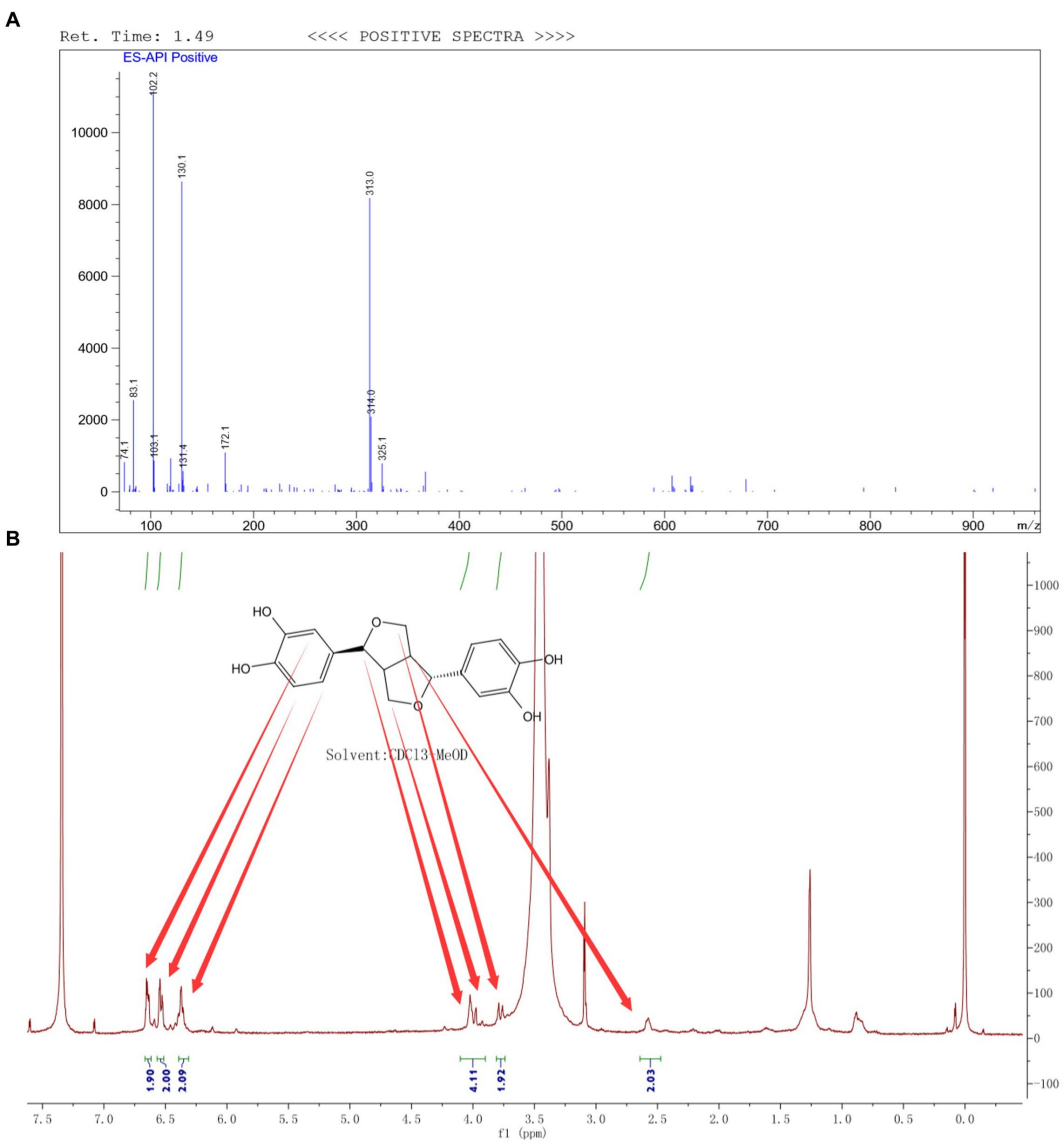
Identification of PHI-Der. **(A)** Identification of PHI-Der using mass spectrometry. **(B)** Identification of PHI-Der using NMR (hydrogen spectrum).

Through the preparation route (Figure 1A), PHI-Der was successfully prepared with a yield of 45%. The molecular formula of PHI-Der is C_18_H_18_O_6_, and the structural formula of PHI-Der SMILES is OC1 = CC=C(C=C1O)[C@H]1OCC2C1CO[C@H]2C1 = CC=C(O)C(O) = C1. Figure 1B shows the 3D structure of PHI-Der. Figure 2A shows the identification of PHI-Der using mass spectrometry. C_18_H_18_O_6_ ideal characteristic peak was [M]^+^ = 330.33. However, the evident characteristic peak was [*M* −17]^+^ = 313.32. One hydroxyl group had been removed because of the unstable connection. Figure 2B shows the NMR identification (hydrogen spectrum) of PHI-Der: ^1^H NMR (400 MHz, CDCl_3_–MeOD) δ 6.64 (s, 2H), 6.54 (d, *J* = 6.6 Hz, 2H), 6.36 (d, *J* = 6.6 Hz, 2H), 4.03.97 (m, 4H), 3.77 (d, *J* = 12.4 Hz, 2H), and 2.65–2.50 (m, 2H) and deuterium with chloroform–deuterium with the methanol solvent. Table 1 shows the physicochemical properties of PHI-Der, in which the lipid–water partition coefficient (logarithm) is 2.58 log(mol/mol), which is less than 3 log(mol/mol), indicating that PHI-Der has good water solubility. Supplementary Figure S1 shows the Fourier transform infrared analysis image of PHI-Der. Supplementary Figure S2 shows the NMR identification (carbon spectrum) of PHI-Der.

**Table 1:** Physicochemical properties of PHI-Der.

Name	Numerical value
Molecular weight	330.34 g/mol
Heavy atoms	24
Aromatic heavy atoms	12
Fraction Csp3	0.33
Rotatable bonds	2
H-bond acceptors	6
H-bond donors	4
Ring count	4
Aromatic ring count	2
Molar refractivity	83.91 m^3^/mol
Topological polar surface area (TPSA)	99.38 Å^2^
Lipid-water partition coefficient (log)	2.58 log(mol/mol)
Acid dissociation constant (pKa)	8.32 log(mol/mol)
Water solubility (log)	−3.75 log(mol/L)

### 3.2. *In vitro* antibacterial activity of PHI-Der against *Helicobacter pylori*

**Figure 3:**
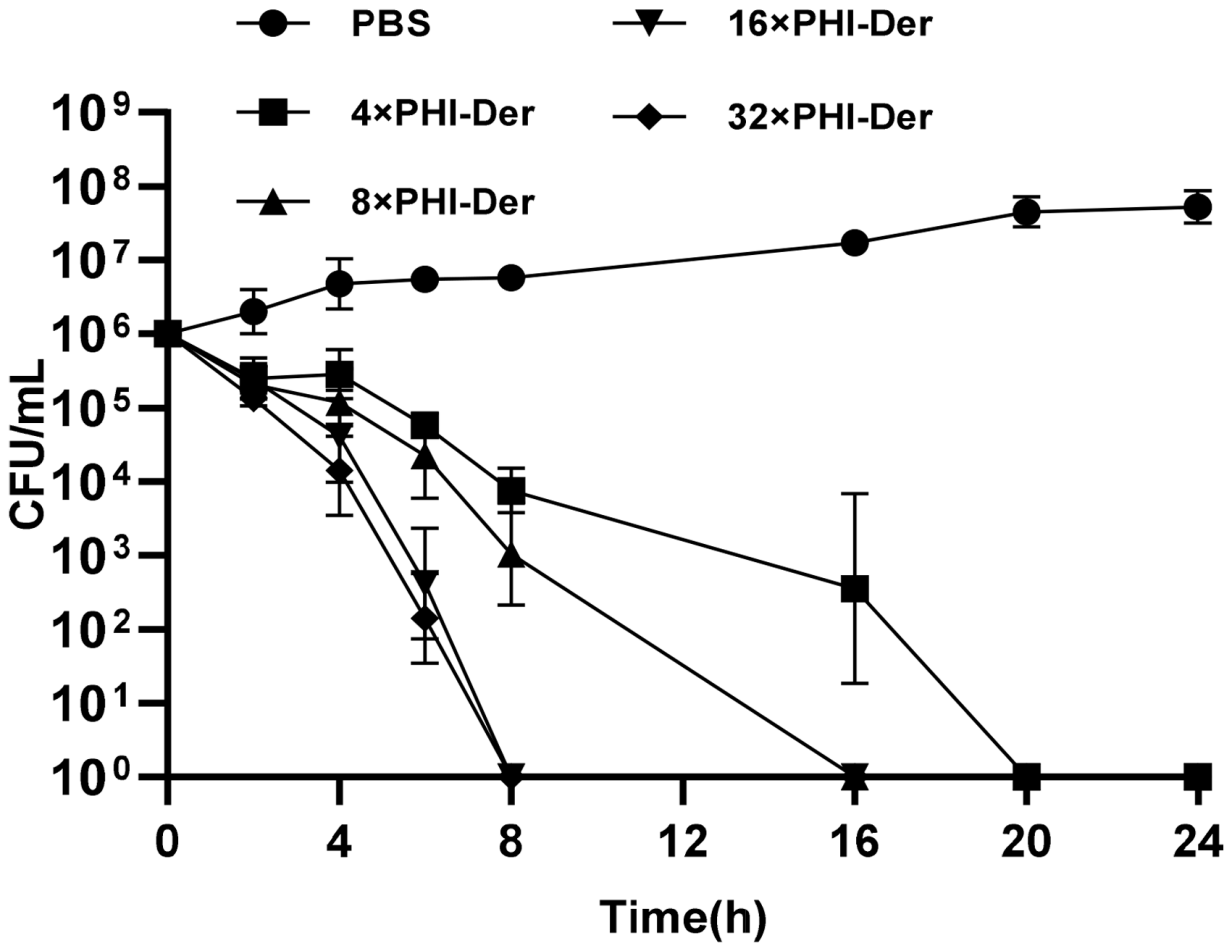
MBC of PHI-Der on *H. pylori.* 4×, 8×, 16×, 32× means the concentration tested in terms of MIC.

PHI-Der MIC was 8–32 μg/mL in 18 *H. pylori* strains (Table 2). PHI-Der exerted better inhibitory effects on sensitive and resistant strains compared to phillygenin. The MBC of PHI-Der against *H. pylori* was 16 times the PHI-Der MIC, reaching 99.9 and 99.999% after 6 and 8 h, respectively. The antibacterial rate was 8 times the PHI-Der MIC, reaching 90, 99, and 99.9% after 4, 6, and 8 h, respectively. The bactericidal effect was related to the concentration and time (Figure 3).

**Table 2:** MICs of PHI-Der against *H. pylori* (μg/mL).

Strain	Drug resistance	PHI-Der	Phillygenin
26695	Sensitive	16	32
HPBS001	Sensitive	16	16
G27	Sensitive	16	16
HPBS002	Sensitive	16	32
HPBS003	Resistant to LEV, CLA, and MET	16	16
HPBS004	Resistant to MET	16	16
HPBS005	Resistant to CLA	16	16
HPBS006	Resistant to LEV	16	32
HPBS007	Resistant to LEV and LEV	8	64
HPBS010	Resistant to CLA and MET	16	16
HPBS011	Resistant to CLA	16	32
HPBS012	Resistant to MET, CLA, and LEV	8	16
HPBS013	Resistant to MET and CLA	16	16
HPBS014	Sensitive	32	64
HPBS015	Resistant to MET, CLA, and LEV	16	16
HPBS016	Resistant to MET, CLA, AMO, and LEV	16	16
MSD132	Sensitive	8	16
NSH57	Sensitive	16	16

### 3.3. Antibacterial activity of PHI-Der against *Helicobacter pylori in vivo*

**Figure 4:**
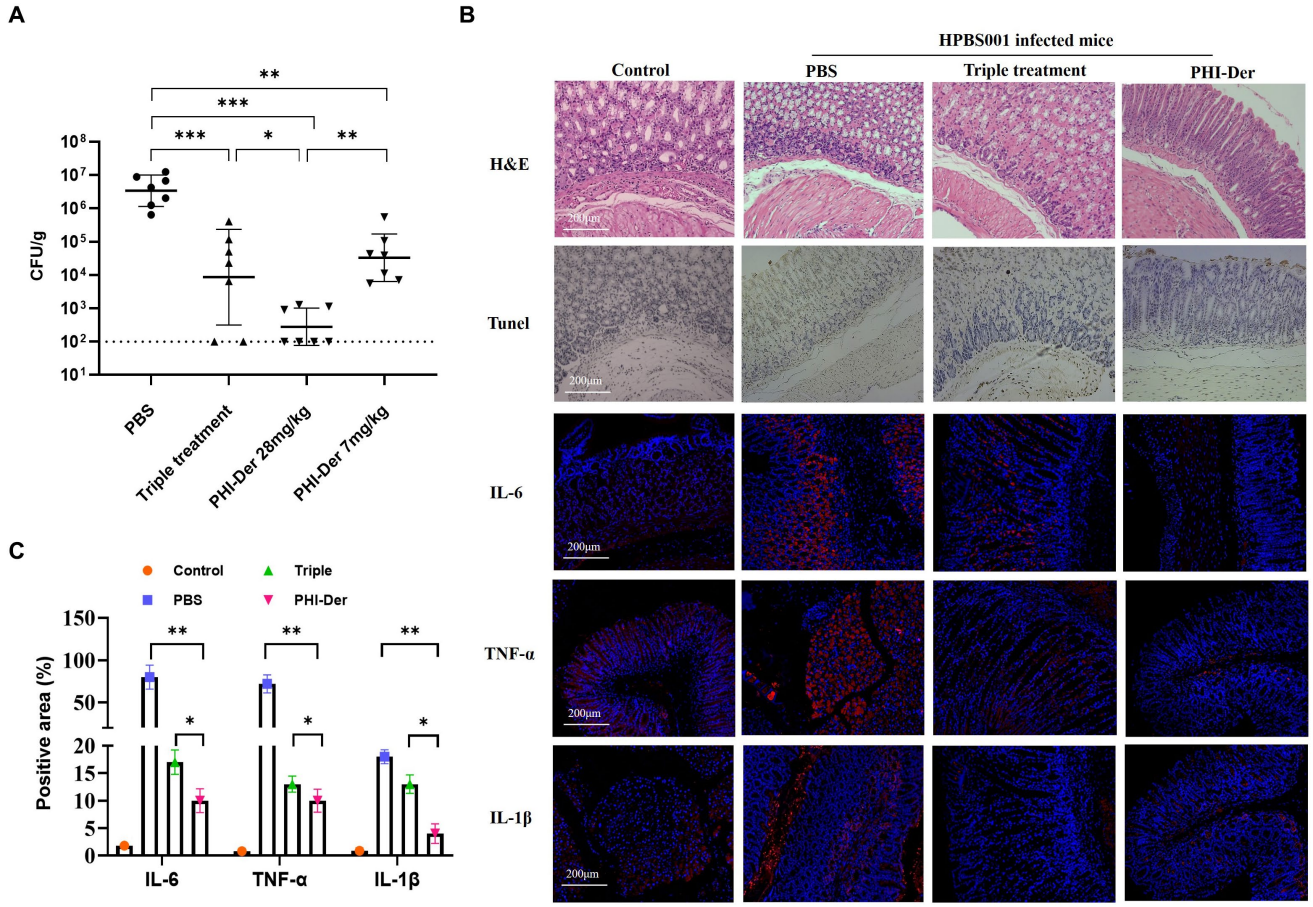
Antibacterial effect of PHI-Der in mice. **(A)** HPBS001 colonization amount in mice with acute gastritis. **(B)** Gastric mucosal tissue repair and inflammatory response in mice with acute gastritis (100 times). **(C)** Quantification of inflammatory factors. *p < 0.05, **p < 0.01, ***p < 0.001.

The efficacy of PHI-Der against *H. pylori in vivo* was assessed using the C57BL/6 mouse model infected with *H. pylori* (HPBS001; Supplementary Figure S3). Based on the counted number of colonies, the bacteriostatic effect of PHI-Der was better than that of the triple therapy. Further, the bacteriostatic effect at high PHI-Der concentrations was better than that at low PHI-Der concentrations (Figure 4A). According to the H&E staining and immunohistochemical images of the PHI-Der group, apoptotic cells and inflammatory factors in the gastric mucosa reduced significantly (Figure 4B). As for the expression of inflammatory factors in tissue samples, expression levels of interleukin-6, tumor necrosis factor-α, and interleukin-1β were the lowest in the PHI-Der group (Figures 4B,C), use ImageJ for quantification. PHI-Der exerted good bacteriostatic effects *in vivo*.

### 3.4. Biosafety of PHI-Der

**Figure 5:**
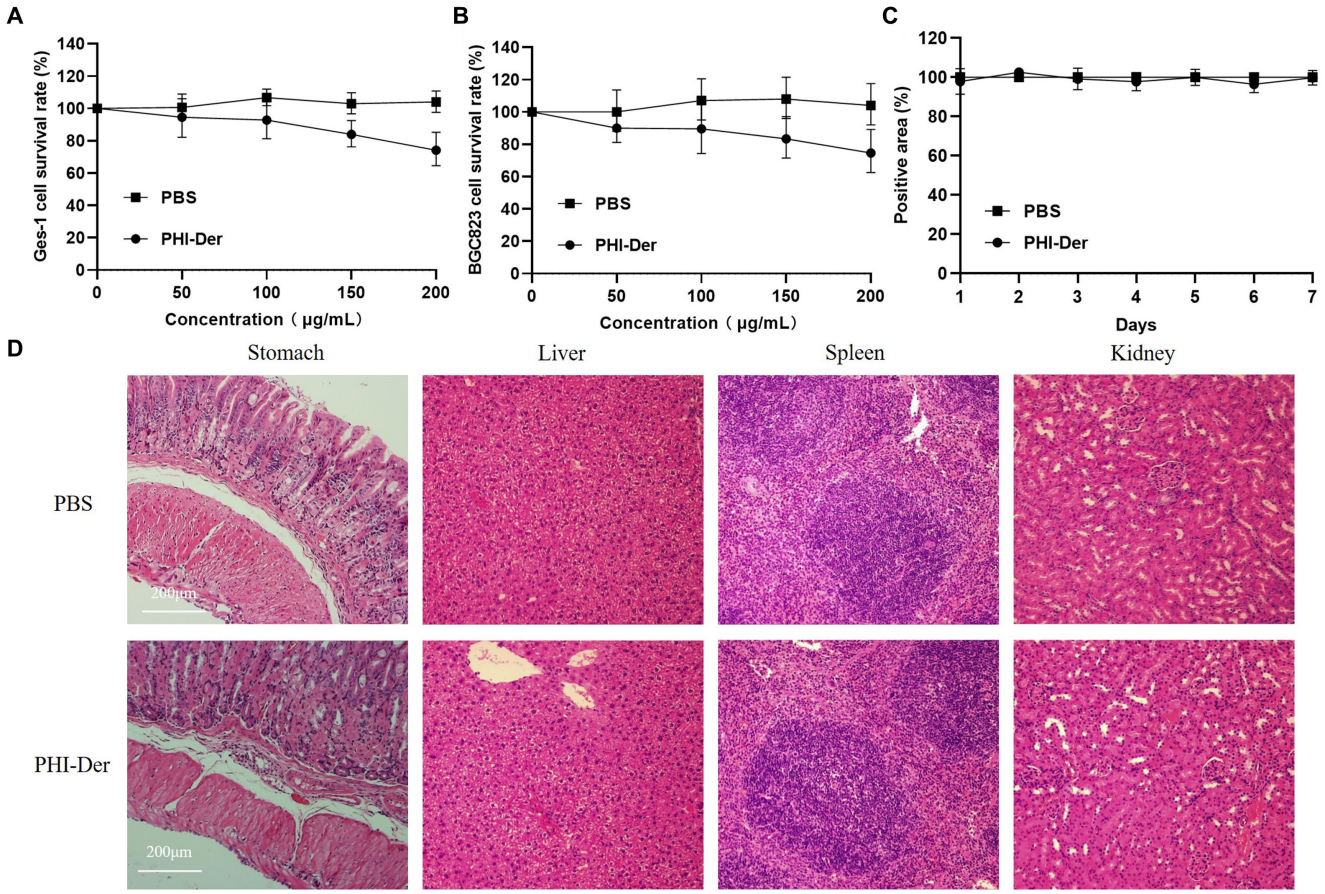
PHI-Der toxicity assay. **(A)** PHI-Der on GES-1 cytotoxicity. **(B)** PHI-Der on BGC823 cytotoxicity. **(C)** PHI-Der effect on the body weight of mice. **(D)** Damage detection of PHI-Der in the stomach, liver, spleen, and kidney of mice (100 times).

The toxicity test of PHI-Der showed that PHI-Der at 100 μg/mL exerted no cytotoxic effect on GES-1 gastric epithelial or BGC823 gastric cancer cells, and the survival rates were above 90% (Figures 5A,B). After the intragastric administration of 10 times the therapeutic dose of PHI-Der, the body weight showed no significant change within 7 days (Figure 5C). The stomach, liver, spleen, or kidney showed no pathological damage (Figure 5D). Pathology scores were in Supplementary Table S3. PHI-Der had low toxicity *in vitro* and *in vivo* and high safety and could be used as first-line drugs to treat *H. pylori*.

### 3.5. Antimicrobial spectrum and drug resistance of PHI-Der

A total of 20 non-*H. pylori* strains were detected, and MICs were above 128 μg/mL. PHI-Der could act on *H. pylori* alone (Table 3) as a narrow-spectrum antibacterial, with specific effects on other bacteria.

**Table 3:** MICs (μg/mL) of PHI-Der against non-*H. pylori.*

Strain	Drug resistance	PHI-Der
*Proteus mirabilis*	Sensitive	>128
*Cryptococcus neoformans*	Resistance	>128
*Candida tropicalis*	Sensitive	>128
*Campylobacter*	Sensitive	>128
*Bacillus subtilis*	Sensitive	>128
*Morganella morganii*	Sensitive	>128
*Staphylococcus haemolyticus*	Sensitive	>128
*Stenotrophomonas maltophilia*	Sensitive	>128
*Acetobacter pasteurianus*	Sensitive	>128
*Escherichia coli*	Sensitive	>128
*Lactobacillus curvatus*	Sensitive	>128
*Saccharomyces cerevisiae* Hansen	Sensitive	>128
*B. fragilis*	Sensitive	>128
*Bifidobacterium longum*	Sensitive	>128
*Enterobacter hormaechei*	Sensitive	>128
*Staphylococcus aureus*	Methicillin-resistant	>128
*Candida Albicans*	Sensitive	>128
*Klebsiella pneumoniae*	Sensitive	>128
*Pseudomonas aeruginosa*	Sensitive	>128
*Acinetobacter baumannii*	Sensitive	>128

**Figure 6:**
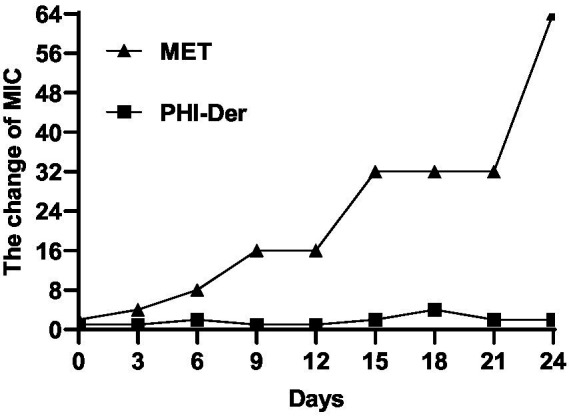
Resistance detection of PHI-Der.

In the 24-day drug resistance induction detection of PHI-Der against *H. pylori* G27 strains, the MIC of PHI-Der had changed only two folds on day 6 and occurred on day 12. However, the MIC of metronidazole increased by 64 times (Figure 6). Resistance to PHI-Der was difficult.

### 3.6. Prediction of targets of PHI-Der

A total of 12 genes were screened using SwissTargetPrediction to predict the targets of PHI-Der (Supplementary Table S4). The target interaction relationship was obtained from the STRING database. Supplementary Figure S4 shows that the interaction relationship between the targets was not close, and targets and pathways were multiple. Targets related to “*H. pylori* infection” were screened using the GeneCards database, and disease targets were collected, including four targets that were repeated with PHI-Der (Table 4).

**Table 4:** PHI-Der act on *H. pylori* infection targets.

Target	Common name	Uniprot ID	ChEMBL ID	Target class
Arachidonate 5-lipoxygenase	ALOX5	P09917	CHEMBL215	Oxidoreductase
Induced myeloid leukemia cell differentiation protein Mcl-1	MCL1	Q07820	CHEMBL4361	Other cytosolic protein
PI3-kinase p110-alpha subunit	PIK3CA	P42336	CHEMBL4005	Enzyme
Serotonin transporter	SLC6A4	P31645	CHEMBL228	Electrochemical transporte

### 3.7. Inhibitory effect of PHI-Der on *Helicobacter pylori* biofilm

**Figure 7:**
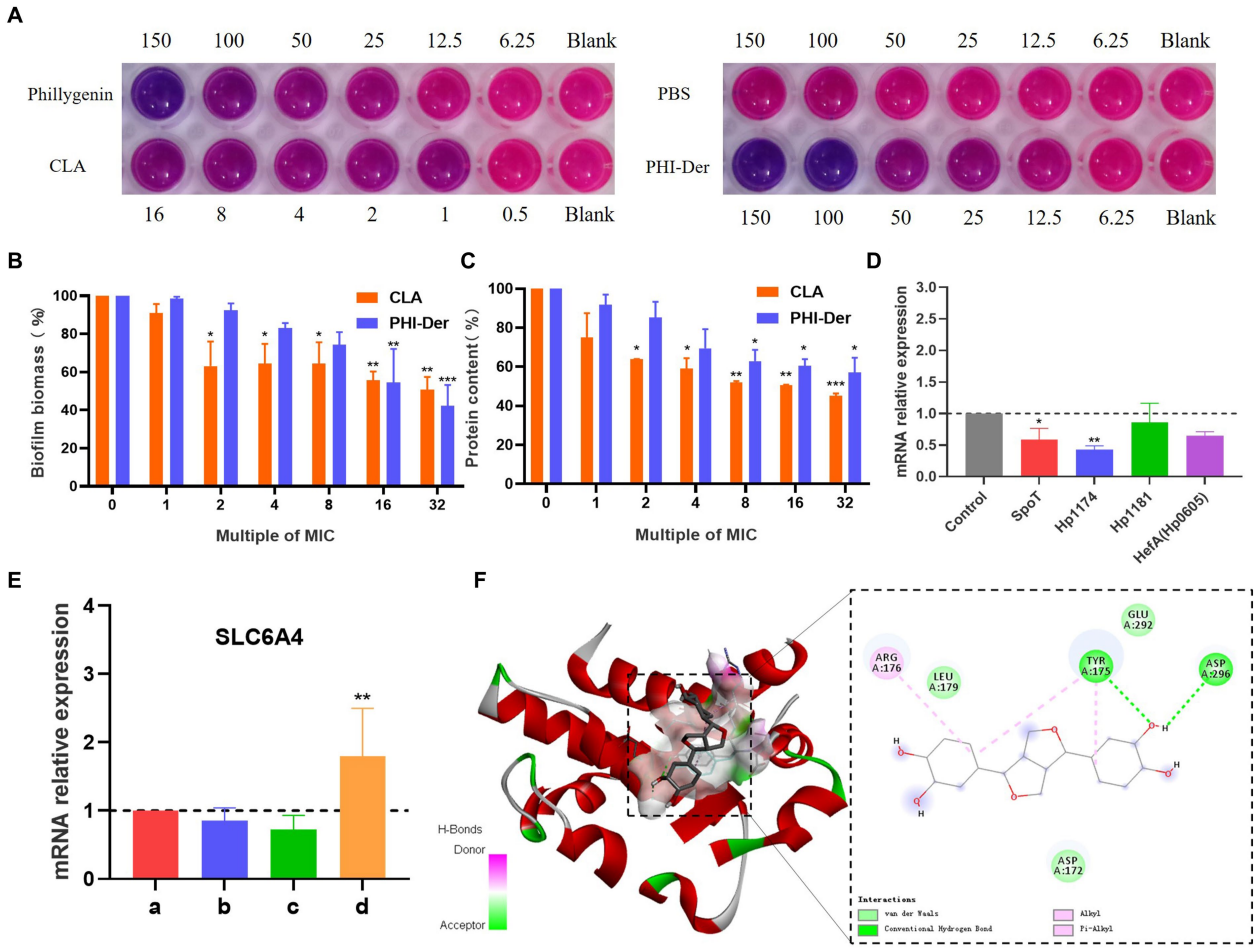
Inhibitory effect of PHI-Der on biofilms. **(A)** Alpha blue showing biofilm inhibition. **(B)** Crystal violet showing biofilm expression. **(C)** Biofilm protein expression. **(D)** Changes in the relative messenger RNA (mRNA) expression of biofilm-related genes after the administration of PHI-Der. **(E)** Changes in the relative expression of *SLC6A4* mRNA after the administration of PHI-Der (a: cell group; b: cells + PHI-Der group; c: infected cell group; d: infected cell + PHI-Der group). **(F)** PHI-Der and *SLC6A4* molecule docking. *p < 0.05, **p < 0.01, ***p < 0.001.

Staining with Alma blue staining solution, pink represents the number of living cells and blue represents the number of dead cells, result showed that PHI-Der at 50–100 μg/mL could effectively inhibit biofilm formation, with a better inhibitory effect than that of PHI(100–150 μg/mL) (Figure 7A). Crystal violet staining showed that 16 times the PHI-Der MIC could inhibit 50% of biofilm formation, which was better than the effect of clarithromycin (Figure 7B). The main component of the extracellular matrix in *H. pylori* biofilms is protein (Pinho et al., 2022), a protein content of biofilm 8–16 times the PHI-Der MIC could inhibit 50% of the biofilm formation, consistent with the results of crystal violet and Alma blue staining (Figure 7C).

The expression of biofilm-related genes (Cai et al., 2020; Spiegel et al., 2021; Pinho et al., 2022) was detected (Figure 7D). *SpoT* is a bifunctional enzyme with the properties of guanosine tetraphosphate (p-ppGpp) synthase and hydrolase (Hathroubi et al., 2017), *Hp1174* is a gene of the major facilitator superfamily (MFS) efflux pump family (Xiaoran et al., 2018), PHI-Der could downregulate *SpoT* and *Hp1174*, indicating that it could inhibit *H. pylori* biofilms through these genes. The serotonin transporter (*SLC6A4*) indirectly regulated the formation of extracellular polymeric substances (EPSs) that induce functional gastrointestinal diseases (Arisawa et al., 2012). The *SLC6A4* expression was upregulated after PHI-Der acted on infected cells (Figure 7E). PHI-Der was docked with SLC6A4 molecules (Figure 7F). The binding energy of docking was −6.6 kcal/mol, which is less than −5 kcal/mol, indicating that PHI-Der can stably bind to the cavity of the SLC6A4 protein. It can separate from amino acids, such as TYR175, LEU179, and ARG176, in the protein. The formation of hydrogen bonds, van der Waals forces, and Pi-Alkyl/Alkyl bond interactions enable PHI-Der to bind to the main active site of the SLC6A4 protein.

### 3.8. Effects of PHI-Der on *Helicobacter pylori* oxidation (ROS production)

**Figure 8:**
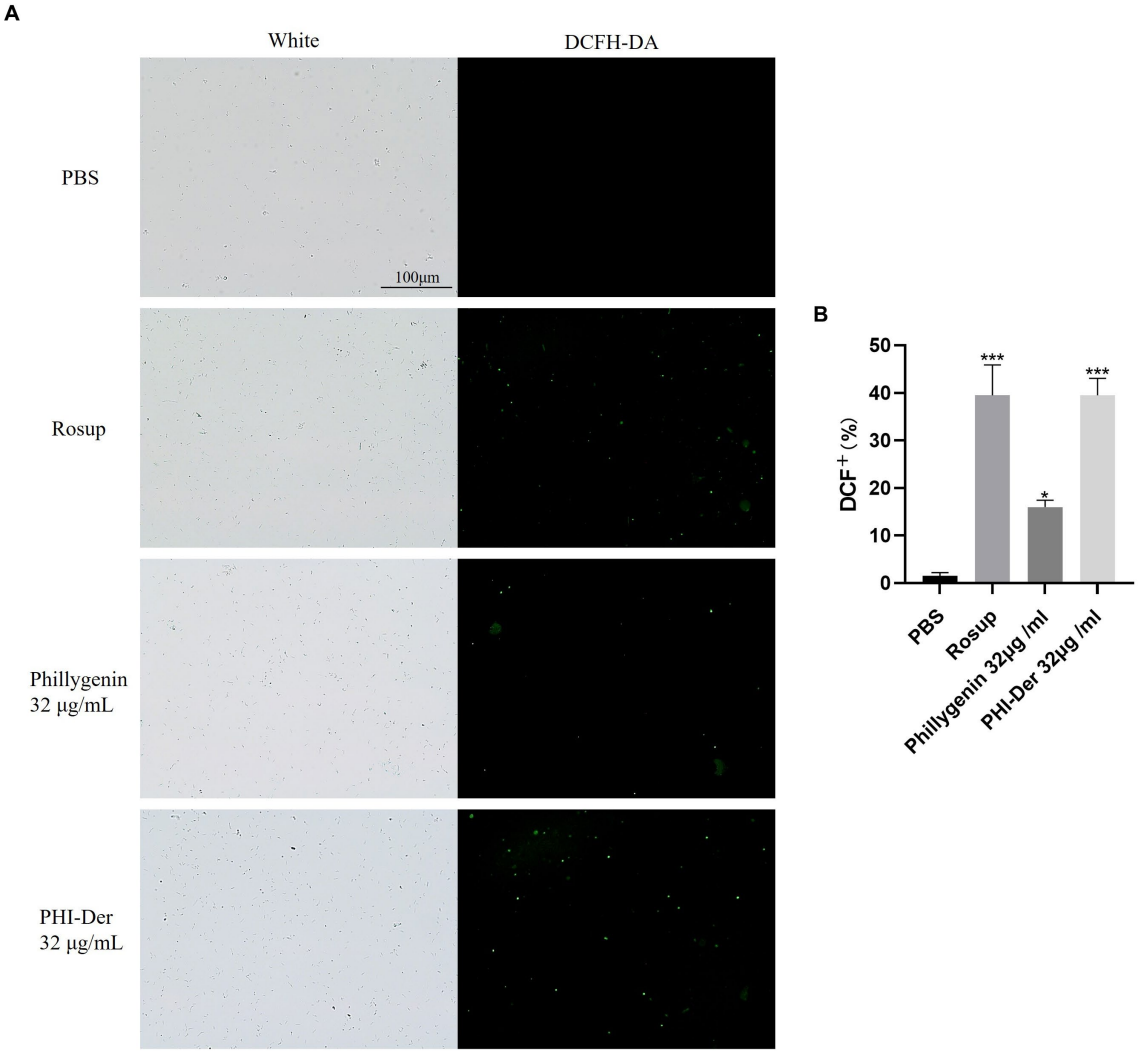
**(A)** Effect of PHI-Der on the oxidation (ROS production) of *H. pylori*. **(B)** Quantitative diagram of the oxidative (ROS production) effect. *p < 0.05, **p < 0.01, ***p < 0.001.

After the drug penetrates into *H. pylori*, it may undergo redox reactions with proteins, nucleic acids, lipids, etc., and produce some peroxides, such as hydrogen peroxide, which can decompose DCFH-DA into dichlorofluorescein yellow and generate fluorescence. At a concentration of 32 μg/mL, the oxidation reaction effect of PHI-Der was better than that of phillygenin (Figures 8A,B).

### 3.9. Antioxidative effect of PHI-Der on infected GES-1

**Figure 9:**
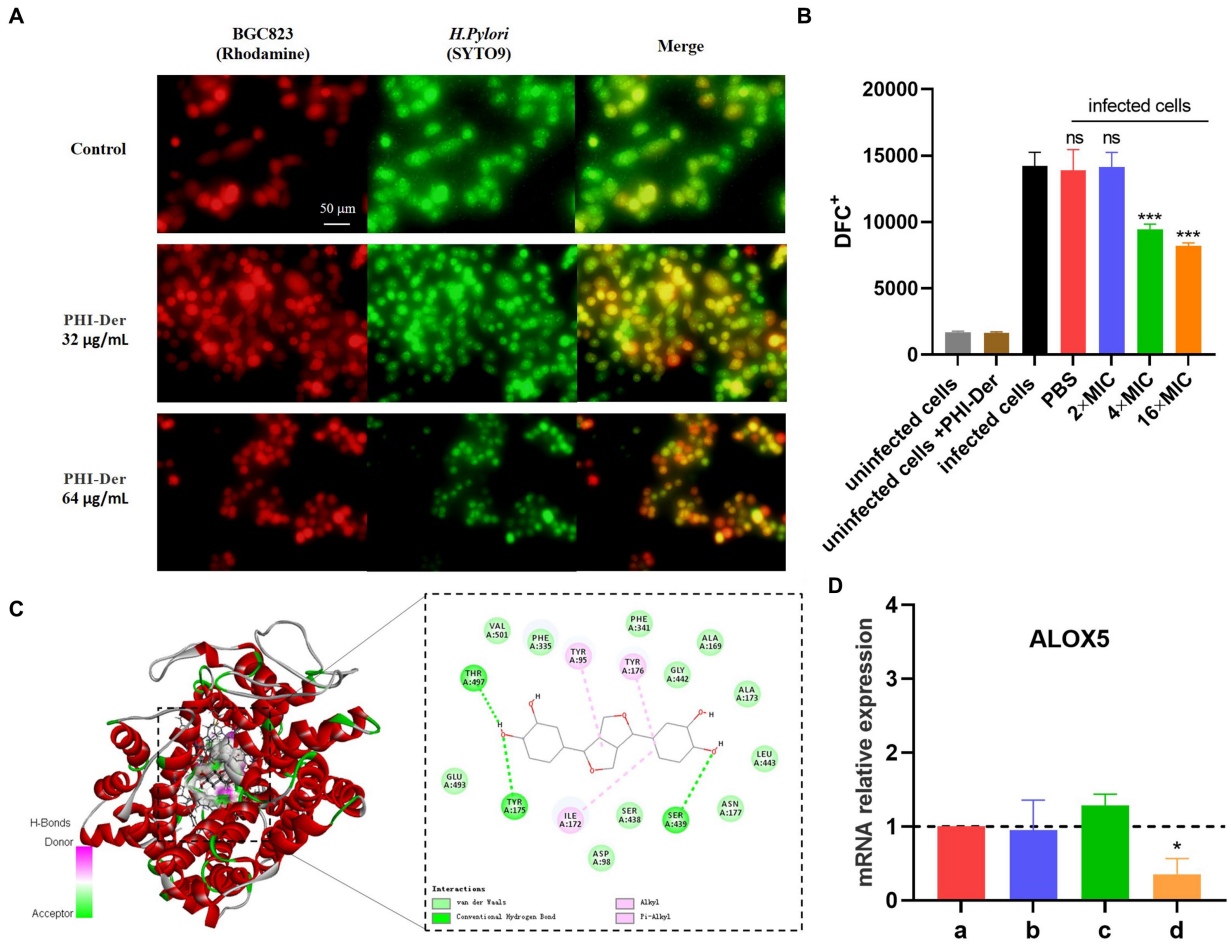
Antioxidative effect of PHI-Der on infected GES-1. **(A)** PHI-Der preventing bacteria from infecting cells. **(B)** Antioxidative effects of PHI-Der on infected GES-1 cells. **(C)** Changes in the relative expression of *ALOX5* mRNA after the action of PHI-Der (a: cell group; b: cell + PHI-Der action group; c: infected cell group; d: infected cell + PHI-Der group). **(D)** PHI-Der was docked with ALOX5 molecules. *p < 0.05, ***p < 0.001.

*Helicobacter pylori* adherence to the cell surface is the first step in infection, which is also a critical step in biofilm formation, with the addition of PHI-Der, the number of *H. pylori* in infecting cells decrease, PHI-Der prevented bacteria from infecting cells (Figure 9A). The activation of inflammatory cells could increase ROS production at the site of inflammation. Without antioxidants, cell function is hindered, and tissue damage occurs, eventually leading to oxidative DNA damage and activation of signaling pathways related to the pathogenesis of gastric cancer (Komatsu et al., 2015). After PHI-Der acted on infected GES-1, the antioxidant effect was not obvious at 2 times the MIC but significantly increased at 4 times the MIC, showing a dose-dependent effect (Figure 9B). PHI-Der could exert an antioxidant effect on GES-1 infected with *H. pylori.* Arachidonic acid lipoxygenase 5 (ALOX5), a key enzyme that mediates lipid peroxidation by producing lipid peroxides, plays a central regulatory role in inflammation (Tang et al., 2021). The *ALOX5* expression was downregulated after PHI-Der acted on infected cells (Figure 9C). PHI-Der was docked with ALOX5 molecules (Figure 9D). The binding energy of docking was −9.9 kcal/mol, indicating that PHI-Der could stably bind to the compound in the cavity of the ALOX5 protein and interact with it. Amino acids, such as THR497, VAL501, and TYR95, formed hydrogen bonds, van der Waals forces, and Pi-Alkyl/Alkyl bond interactions, which enabled PHI-Der to bind to the main active site of the ALOX5 protein.

## 4. Discussion

The drug resistance of *H. pylori* has been increasing yearly. An effective way to prepare novel antibacterial drugs is to find active ingredients from natural products (natural plants, microbial secondary metabolites, marine organisms) and modify and transform them into derivatives (Shen et al., 2020). In traditional Chinese medicine, the active ingredient phillygenin was screened from *Forsythia*, which belongs to the category of diepoxy lignans. As a type of natural aromatic polymer, lignans contain phenolic hydroxyl, alcohol hydroxyl, and carbonyl groups (Westwood et al., 2016). Demethylation modification enhances its molecular reactivity (Huan et al., 2021). PHI-Der had been prepared by removing the methyl group of the ortho-methoxy group from the phenolic hydroxyl group, but this method only removed one methyl group (Liu et al., 2022). In a previous study, the ideal molecular weight of PHI-Der was 330.33; however, mass spectrometry showed a characteristic peak at 313.32, which may be because of the unstable connection of the hydroxyl group (Hannah et al., 2022). Therefore, one hydroxyl group was removed, and the molecular weight of the hydroxyl group was optimally 17, consistent with our speculation.

Due to the complexity of strains of infectious diseases, multiple subtypes of pathogenic bacteria might cause the same disease (Xunlei et al., 2019). To evaluate the antibacterial activity of PHI-Der against different *H. pylori* strains, we randomly selected 18 *H. pylori* strains with different sources and sensitivities. After testing, the MIC of PHI-Der was 8–32 μg/mL. It had the same effect on sensitive and resistant *H. pylori* strains, with an antibacterial effect 2–8 times better than that of phillygenin. Antibacterial rates were 90, 99, and 99.9% under the action of 8 times the MIC for 4, 6, and 8 h, respectively. Thus, sterilization was related to concentration and time. The efficacy was evaluated *in vitro*, and the MIC of PHI-Der against 20 non-*H. pylori* strains was detected. It had the characteristics of specific inhibition of *H. pylori*. The CCK-8 cytotoxicity test showed that the survival rates of GES-1 and BGC823 cells were above 90% when the PHI-Der concentration was 100 μg/mL. In addition, the mice were administered with 10 times the therapeutic dose *via* gavage, and no organ damage was found. Thus, both *in vivo* and *in vitro* experiments proved that PHI-Der was relatively safe. In the *in vivo* evaluation of drug efficacy, the effect of PHI-Der was better than that of the triple therapy. After treatment with PHI-Der, apoptotic cells were reduced, and inflammation was alleviated. PHI-Der was effective against drug-resistant strains *in vivo*, with a better therapeutic effect. A certain PHI-Der concentration had a good therapeutic effect on refractory gastritis caused by clinical drug-resistant *H. pylori* infection. PHI-Der could become a leading drug or candidate drug against *H. pylori*.

Bacterial biofilms are bacterial communities located in self-assembled matrices called EPS, which are mainly composed of proteins (Hou et al., 2022). After formation, biofilms serve as a sanctuary for bacteria to resist antibiotic treatment and immune defense, thereby causing drug resistance (Krzyżek et al., 2022). *Hp1174*, a gene of the major facilitator superfamily efflux pump family, is involved in biofilm formation. In this study, PHI-Der could effectively inhibit biofilm formation, with a better effectiveness than that of phillygenin. Its mechanism is related to downregulation of *Hp1174* expression. The serotonin transporter (*SLC6A4*) is associated with functional dyspepsia in *H. pylori* infection (Hwang et al., 2014). miR-325 regulates and induces the formation of functional gastrointestinal disease EPS and is present in *SLC6A4.* At a strong binding site, miR-325 expression is attenuated upon binding (Arisawa et al., 2012). In this study, *SLC6A4* was significantly upregulated after the effect of PHI-Der, which indicated that EPS formation was weakened and that biofilm formation was inhibited. In target prediction, PHI-Der could regulate *SLC6A4*, consistent with the phenotype of inhibiting biofilm formation. The inhibitory effect of demethylated hydroxylated PHI-Der on biofilm is better than that of phillygenin (Li et al., 2022), which may be because of the fact that the hydroxyl group attached to anthraquinone can target and regulate *SLC6A4* and *Hp1174*, attenuating miR-325 expression, thereby inhibiting EPS formation more effectively (Song et al., 2021).

*Helicobacter pylori* promotes persistent inflammation, thereby maintaining a microenvironment rich in cytokines/chemokines, reactive nitrogen species, and ROS, which destabilize normal cellular homeostasis (Maciej et al., 2021). PHI-Der can prevent bacteria from infecting cells and cells from oxidizing and exert a protective effect on *H. pylori*-induced GES-1 cells. *ALOX5* can regulate cell death in two ways: inflammation and lipid peroxidation. Excessive lipid peroxidation easily occurs in phospholipids, the main component of plasma membrane, leading to membrane rupture and cell death (Sun et al., 2019). *ALOX5* expression is upregulated after *H. pylori* infection and downregulated after the action of PHI-Der. PHI-Der can regulate inflammation and lipid peroxidation by mediating *ALOX5*, thereby reducing the inflammatory response of infected cells. Thus, cells receive a certain protective effect.

The oxidation of PHI-Der because of *H. pylori* was significantly enhanced, but the mechanism of its action remains unknown. Further, the reason behind enhanced oxidation of *H. pylori* and weakened oxidation of infected cells is unknown. Owing to the increase in hydroxyl groups, oxidative properties of drugs increase (Gao et al., 2020); however, substances that are oxidized differ significantly between prokaryotic and eukaryotic cells. In *H. pylori*, *ALOX5* was not found. In contrast, no *H. pylori*-associated oxidized proteins may be present in eukaryotic cells. This issue needs to be further explored.

In addition, PHI-Der could also regulate ATP leakage of *H. pylori* (Arya et al., 2019) and downregulate virulence factors (Xia et al., 2022) (see Supplementary materials for details). Some pathogens that have evolved virulence factors highly resistant to oxidative stress can adhere and form biofilms on cell surfaces. Therefore, PHI-Der can reduce the oxidative damage of infected cells by inhibiting biofilm formation and the expression of virulence factors, enhancing the oxidation reaction in *H. pylori*, thereby achieving better killing and protection of *H. pylori* and protecting the gastric mucosa.

## 5. Conclusion and outlook

This study showed that demethylated hydroxylated PHI-Der was more effective than phillygenin in treating *H. pylori* infection, with advantages of low toxicity, less likelihood of drug resistance, and specific action on *H. pylori* with better medicinal properties, it’s a good antioxidant for host cells. Chemical modification of demethyl hydroxylation enhanced oxidation and inhibited biofilm formation, which could help modify compounds for improved activity, it provide a new approach for improving the activity of the compound.

## Data availability statement

The original contributions presented in the study are included in the article/Supplementary material, further inquiries can be directed to the corresponding authors.

## Ethics statement

The animal study was reviewed and approved by SYXK Gui 2017-0004.

## Author contributions

R-JL was responsible for the experimental research, performed to consult literature and write the first draft. J-yX, XUW, L-jL, XIW, PX, W-yX, Z-yX, S-hX, Y-yJ, and LH writing—review and editing. L-yW, G-rH, and Y-QH designed, checked and modified finalize the manuscript. All authors contributed to the article and approved the submitted version.

## Funding

This study was supported by National Natural Science Foundation of China, Nos. 81760739 and 32060018 and through special fund projects for Guide Local Science and Technology Development by the China Government (GUIKEZY20198004).

## Conflict of interest

The authors declare that the research was conducted in the absence of any commercial or financial relationships that could be construed as a potential conflict of interest.

## Publisher’s note

All claims expressed in this article are solely those of the authors and do not necessarily represent those of their affiliated organizations, or those of the publisher, the editors and the reviewers. Any product that may be evaluated in this article, or claim that may be made by its manufacturer, is not guaranteed or endorsed by the publisher.

## Footnotes

^1^ https://paddlehelix.baidu.com/

^2^ http://www.Swisstargetprediction.ch/

^3^ https://STRING-db.org/

^4^ http://www.rcsb.org/

## Supplementary material

The Supplementary material for this article can be found online at:

https://www.frontiersin.org/articles/10.3389/fmicb.2023.1071603/full#supplementary-material

## Abbreviations

LEV: levofloxacin
CLA: clarithromycin
MET: metronidazole
AMO: amoxicillin
