# Supplementary material for: Therapeutic effect of demethylated hydroxylated phillygenin derivative on Helicobacter pylori infection
Source: Front Microbiol. 2023 May 19;14:1071603. doi: 10.3389/fmicb.2023.1071603 (PMC10235509; doi:10.3389/fmicb.2023.1071603)
Supplement: Supplementary file 1 [file Data_Sheet_1.docx]

Table S1 Strain information

| Strains | Origin | Medium composition | Culture temperature |
| --- | --- | --- | --- |
| Proteus mirabilis | Clinical separation | Nutrient Agar/ Broth | 37℃ |
| Cryptoccus neo formans | Clinical separation | Sabourauds | 30℃ |
| Candida tropicalis | Clinical separation | Sabourauds | 37℃ |
| Campylobacter | Clinical separation | Columbia Agar Base / Brain-Heart Infusion Broth（With 10% serum） | 37℃ |
| Bacillus subtilis | Clinical separation | Sabourauds | 37℃ |
| Morganella morganii | Guangdong Microbial Culture Collection Center | Nutrient Agar/ Broth | 37℃ |
| Staphylococcus haemolyticus | Clinical separation | Nutrient Agar/ Broth | 37℃ |
| Stenotrophomonas maltophilia | Clinical separation | Nutrient Agar/ Broth | 30℃ |
| Acetobacter pasteurianus | Guangdong Microbial Culture Collection Center | Glucose、Radix scutellariate、Calcium carbonate、Ethanol absolute、Distilled water、Agar（No need to liquid medium） | 30℃ |
| Escherichia coli | Clinical separation | Nutrient Agar/ Broth | 37℃ |
| Lactobacillus curvatus | Guangdong Microbial Culture Collection Center | Peptones、Beef Extract、Yeast extract、Glucose、Sodium acetate、Ammonium citrate dibasic、tween-80、Dipotassium hydrogenphosphate、magnesium sulfate heptahydrate、Manganese sulfate heptahydrate、Calcium carbonate、Distilled water、Agar（No need to liquid medium） | 37℃ |
| Saccharomyces cerevisiae Hansen | Guangdong Microbial Culture Collection Center | Peptone、Glucose、Yeast extract、Malt extract、Distilled water、Agar | 30℃ |
| b.fragilis | Guangdong Microbial Culture Collection Center | TSA+5% Defibrinated Sheep Blood | 37℃ |
| Bifidobacterium longum | Guangdong Microbial Culture Collection Center | BactoTM Soytone、Typtone、Yeast extract、Glucose、saline solution、L-Cysteine、0.1%resazurin、Distilled water、Agar（No need to liquid medium） | 37℃ |
| Enterobacterhormaechei | Clinical separation | Nutrient Agar/ Broth | 37℃ |
| Staphalococcus aureus | Clinical separation | Nutrient Agar/ Broth | 37℃ |
| candida Albicans | Clinical separation | Sabourauds | 37℃ |
| Klebsiella pneumoniae | Clinical separation | Nutrient Agar/ Broth | 37℃ |
| Pseudomonas aeruginosa | Clinical separation | Nutrient Agar/ Broth | 37℃ |
| Acinetobacter baumannii | Clinical separation | Nutrient Agar/ Broth | 37℃ |

Table S2 List of primers

| Name | Forward primers | Reverse primers |
| --- | --- | --- |
| 16s | AGGATCAAGGTTTAAGGATT | CTGGAGAGACTAAGCCCTCC |
| GAPDH | GGACCTGACCTGCCGTCTAG | GTAGCCCAGGATGCCCTTGA |
| SpoT | TGTGCAACCTGTCGCTAATC | TTGGGCAAGATGTGGCTAAT |
| Hp1174（gluP） | AAAATGGGCGATAATGCAAG | TCCACATCAGGCAATTTCAA |
| HefA(hp0605) | CTCGCTCGCATGATCGC | CGTATTCGCTCAAATTCCCT |
| Hp1181 | CACGCCAAGCTTGAGTAACA | CAAAGCGGCTTCCAAATAAA |
| ALOX5 | TGGAATGACTTCGCCGACTTTGAG | GCAGCCATTCAGGAACTGGTAGC |
| MCL1 | AGTTTGGGTCTCTGTTGCTATGGC | CCGTTTGAGTTCCTGTCCTGATGTC |
| PIK3CA | ACAGCTCAAAGCAATTTCTACACG | AGCACTTACCTGTGACTCCATAG |
| SLC6A4 | TCGTAGTCGTAACCCACCCATCTG | GAACAGCAGTTGCGGAGAGTCAG |
| Caggamma | AGGCTGCGACAATGAAGTGGTG | CGCGCTTGTTGTTCAACCCTAAAG |
| CagA | ACCCCTAGTCGGTAATG | GCTTTAGCTTCTGATACTGC |
| VagA | GTCAGCATCACACCGCAAC | CTGCTTGAATGCGCCAAAC |


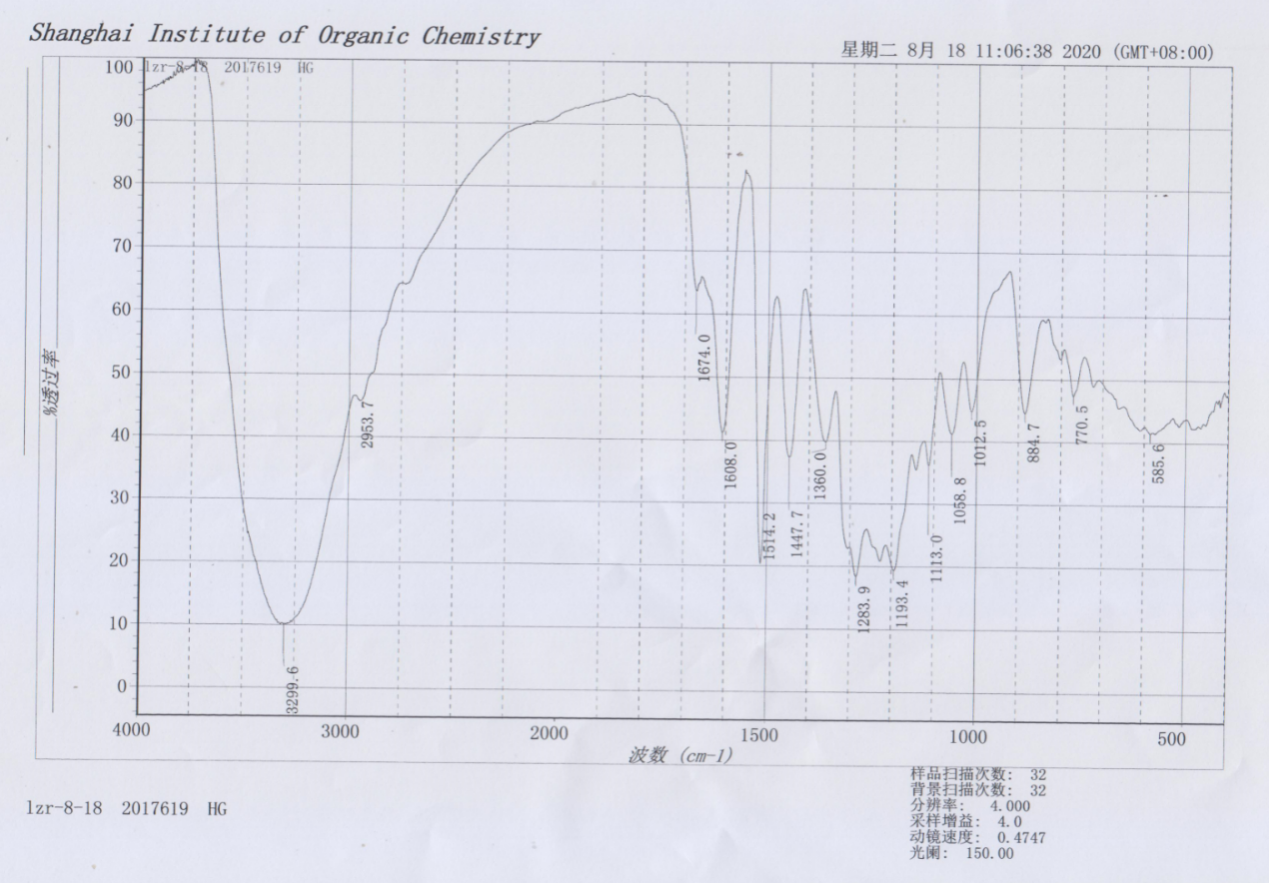


Figure S1. Fourier transform infrared analysis of PHI-Der. 3299 cm^−1^ phenolic hydroxyl stretching vibration; 1360 cm^−1^ phenolic hydroxyl in-plane bending; 1283 cm^−1^ phenolic hydroxyl C–O stretching; 1193 cm^−1^ C–O–C stretching; 884 cm^−1^ 770 cm^−1^ 585 cm^−1^ benzene ring characteristic peak.


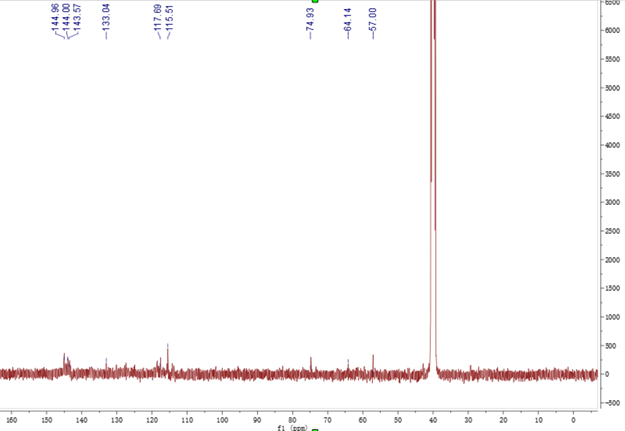


Figure S2. NMR identification (carbon spectrum) of PHI-Der. ^13^C NMR (101 MHz, CDCl_3_–MeOD) δ 144.99 (C–O), 144.00(C–O), 143.57 (C), 133.04 (CH), 117.69 (CH), 115.51 (CH), 74.93 (C–O), 64.14 (CH_2_), 57.00 (CH), deuterium with chloroform–deuterium with the methanol solvent.


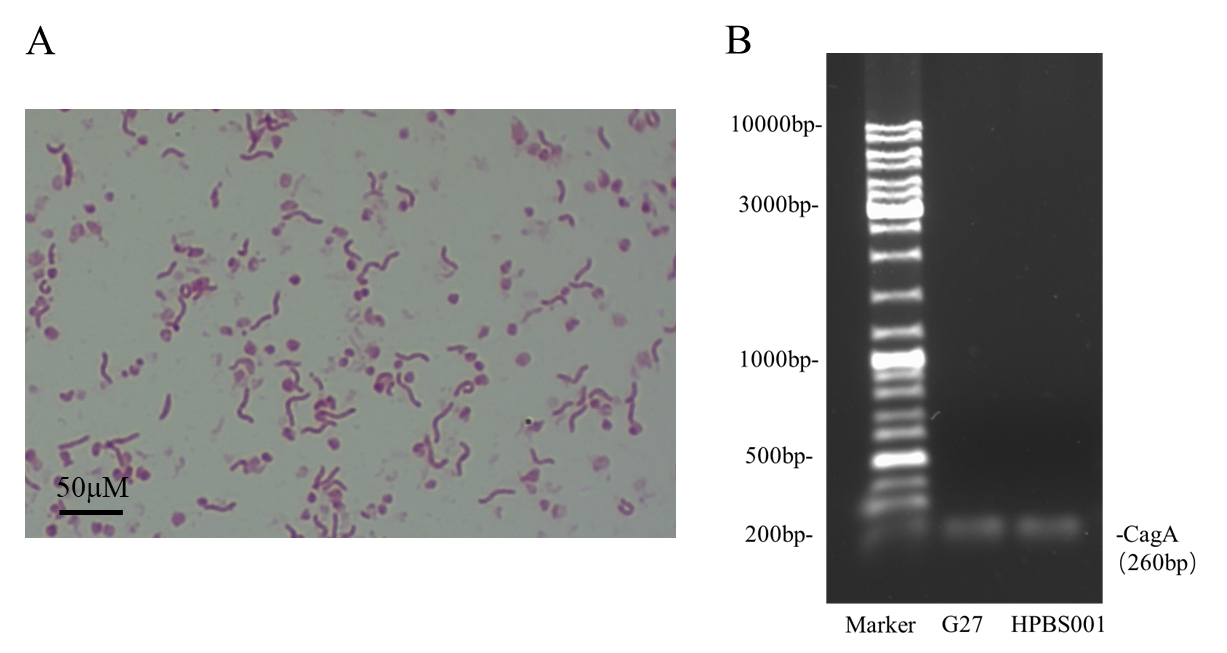


Figure S3. Identification of animal strains. A. Gram staining diagram showing curved and negative bacilli. B. PCR amplification result. Compared to the standard strain, the isolated strain can amplify *CagA*.

Table S3 Pathology scores

| Group | n | stomach | liver | spleen | kidney |
| --- | --- | --- | --- | --- | --- |
| PBS | 5 | 0.3±0.1 | 0.2±0.1 | 0.1±0.1 | 0.3±0.2 |
| PHI-Der | 5 | 0.3±0.2 | 0.3±0.3 | 0.2±0.2 | 0.3±0.4 |
| Value P |  | >0.05 | >0.05 | >0.05 | >0.05 |

Note: 0 means non-existent; <1% means very mild; 1% to 25% means mild; 26%-50% means moderate; 51%-75% means moderate to severe; 76%-100% means severe.

Table S4 Target prediction

| Target | Common name | Uniprot ID | ChEMBL ID | Target Class |
| --- | --- | --- | --- | --- |
| Arachidonate 5-lipoxygenase | ALOX5 | P09917 | CHEMBL215 | Oxidoreductase |
| Platelet activating factor receptor | PTAFR | P25105 | CHEMBL250 | Family A G protein-coupled receptor |
| Induced myeloid leukemia cell differentiation protein Mcl-1 | MCL1 | Q07820 | CHEMBL4361 | Other cytosolic protein |
| PI3-kinase p110-gamma subunit | PIK3CG | P48736 | CHEMBL3267 | Enzyme |
| PI3-kinase p110-alpha subunit | PIK3CA | P42336 | CHEMBL4005 | Enzyme |
| c-Jun N-terminal kinase 2 | MAPK9 | P45984 | CHEMBL4179 | Kinase |
| Sodium/glucose cotransporter 2 | SLC5A2 | P31639 | CHEMBL3884 | Electrochemical transporter |
| Quinone reductase 2 | NQO2 | P16083 | CHEMBL3959 | Enzyme |
| Mu opioid receptor | OPRM1 | P35372 | CHEMBL233 | Family A G protein-coupled receptor |
| Serotonin transporter | SLC6A4 | P31645 | CHEMBL228 | Electrochemical transporte |
| LXR-alpha | NR1H3 | Q13133 | CHEMBL2808 | Nuclear receptor |
| LXR-beta | NR1H2 | P55055 | CHEMBL4093 | Nuclear receptor |


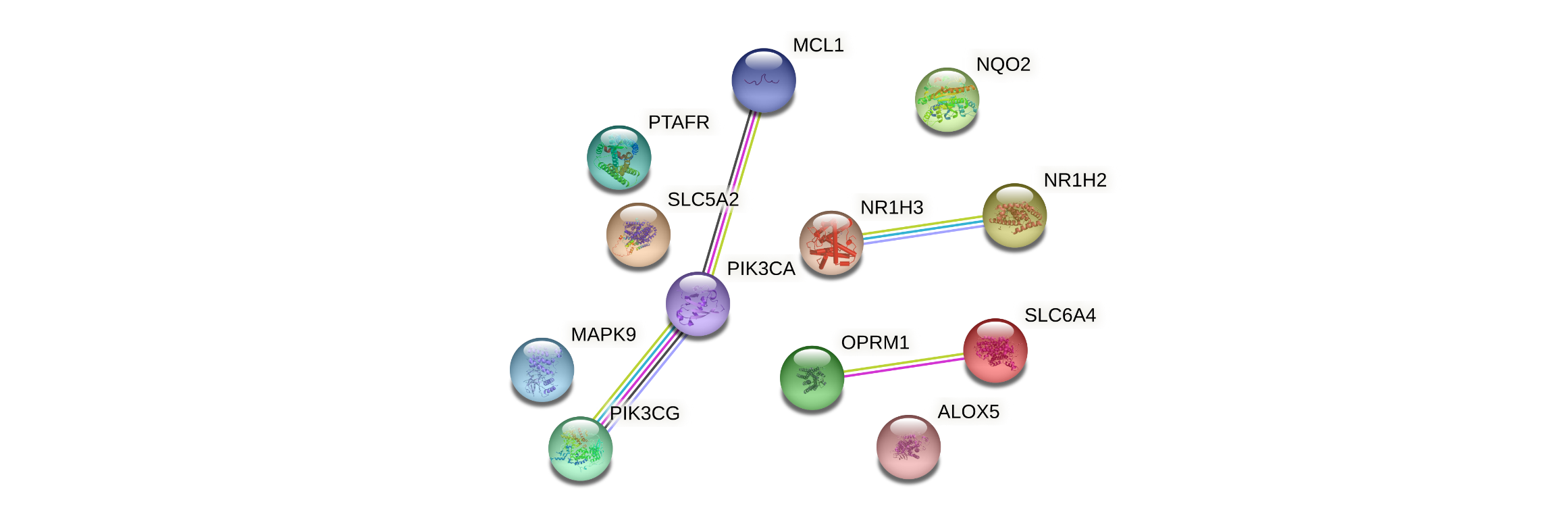


Figure S4 Predicted target protein interaction map


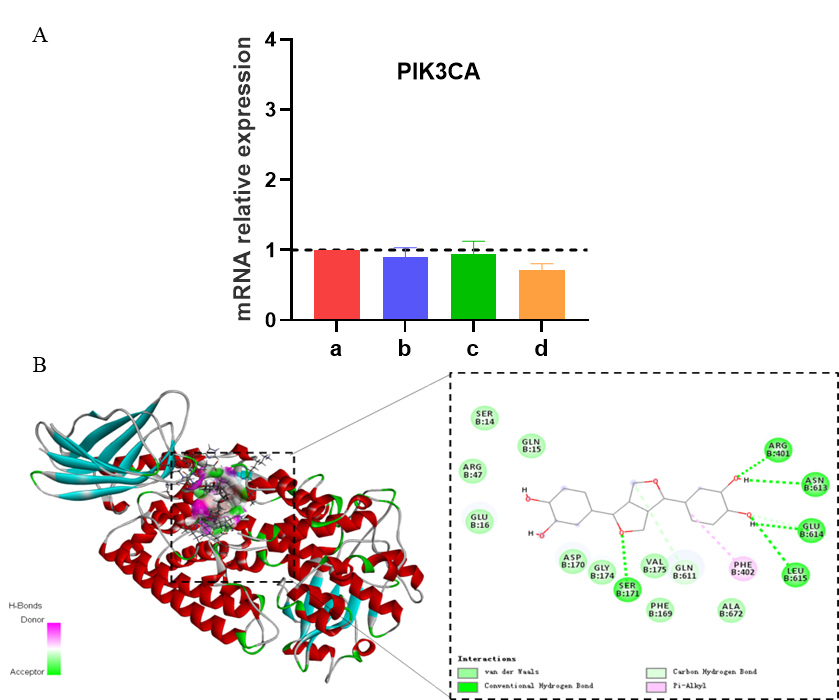


Figure S5. A. Relative expression changes in *PIK3CA* mRNA after the action of PHI-Der (a: cell group; b: cell + PHI-Der action group; c: infected cell group; d: infected cell + PHI-Der group). B. Docking of PHI-Der with *PIK3CA* molecules. **p* < 0.05, ***p* < 0.01, ****p* < 0.001. The binding energy of docking is −7.3 kcal/mol, less than −5 kcal/mol, indicating that PHI-Der can spontaneously compare with the protein. Compounds can stably bind to the cavities of proteins and interact with surrounding amino acids. As shown, compounds interact with proteins primarily through hydrogen bonds, van der Waals forces, and Pi-Alkyl/Alkyl interactions. The compound can form stable hydrogen bonds with AGR401, ASN613, GLU614, LEU615, and SER171 of the catalytic co-protein; the hydrophobic methylene group in the compound can form van der Waals bonds with amino acids, such as SER14, GLN15, AG47, and GLU16 in the protein; the methylene group in the compound can form a conjugated bond with the PHE402 amino acid of the protein to form Pi-Alkyl/Alkyl, which is the main force for the compound to bind to the active site.


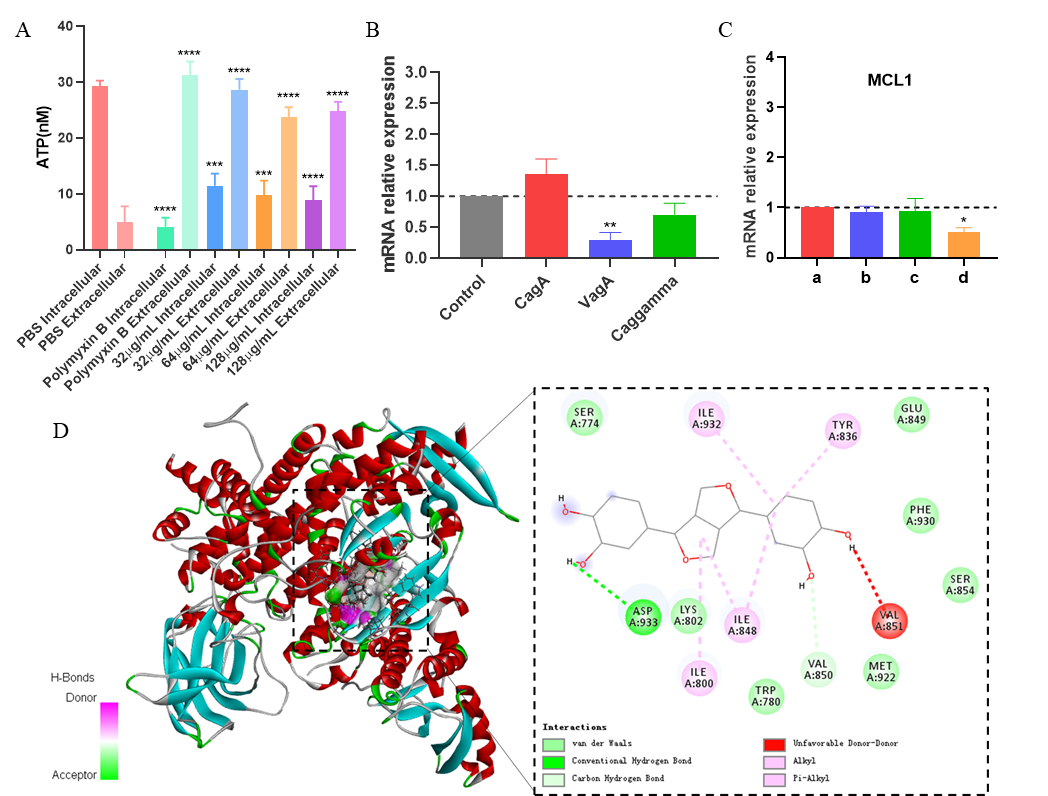


Figure S6. A. ATP detection of *H. pylori* by PHI-Der. *H. pylori* G27 was cultured to the logarithmic phase, and the concentration of the bacterial solution was adjusted to 1×10^7^ CFU/mL, and the working concentration of PHI-Der was set to 32, 64, and 128 μg/mL. Positive control was administered with polymyxin B (Macklin, China), and negative control was administered with PBS. Coculture was performed in a three-gas incubator for 2 h. Centrifugation was performed to obtain the supernatant and bacteria. The supernatant was used as the bacterial extracellular ATP detection sample; 200 μL of lysate was added to the harvested bacteria, and the supernatant was collected using centrifugation after lysis, which was the bacterial intracellular ATP detection sample. The standard curve was determined according to the instructions of the ATP detection kit (Beyotime, China). We added 100 μL of the detection working solution to the sample well, left it at room temperature for 3–5 min, and used a multifunctional microplate reader (BioTek, America) to detect. After 2 h of PHI-Der acting on *H. pylori*, the intracellular ATP gradually leaked into the extracellular space. Most of the ATP was leaked at 2 times the MIC. No difference was found between levels of 4 times the MIC and 8 times the MIC, and most of them leaked. B. Changes in the relative expression of virulence factor mRNA caused by PHI-Der. PHI-Der can significantly downregulate *VagA*. C. Changes in the relative expression of MCL1 mRNA after the effect of PHI-Der (a: cell group; b: cell + PHI-Der action group; c: infected cell group; d: infected cell + PHI-Der action group). PHI-Der was docked with MCL1 molecules, and the binding energy of docking was −7.3 kcal/mol. **p* < 0.05, ***p* < 0.01, ****p* < 0.001.
